# 2357 Lost and found: Detection of brain cardiolipins in plasma after cardiac arrest

**DOI:** 10.1017/cts.2018.91

**Published:** 2018-11-21

**Authors:** Andrew M. Lamade, Tamil S. Anthonymuthu, Elizabeth M. Kenny, Hitesh Gidwani, Nicholas M. Krehel, Andrew A. Amoscato, Adam C. Straub, Valerian E. Kagan, Cameron Dezfulian, Hülya Bayır

**Affiliations:** 1 Department of Critical Care Medicine, School of Medicine, Safar Center for Resuscitation Research, University of Pittsburgh and Children’s Hospital of Pittsburgh, Pittsburgh, PA, USA; 2 Center for Free Radical and Antioxidant Health, Environmental and Occupational Health, Graduate School of Public Health, University of Pittsburgh, Pittsburgh, PA, USA; 3 Department of Pharmacology and Chemical Biology, University of Pittsburgh, Pittsburgh, PA, USA; 4 Vascular Medicine Institute, University of Pittsburgh, Pittsburgh, PA, USA

## Abstract

OBJECTIVES/SPECIFIC AIMS: Neurological injury remains as the main limiting factor for overall recovery after cardiac arrest (CA). Currently available indicators of neurological injury are inadequate for early prognostication after return of spontaneous circulation (ROSC). High diversification of brain mitochondrial cardiolipins (CL) makes them unique candidates to quantify brain injury and to predict prognosis early after ROSC. METHODS/STUDY POPULATION: CL content in plasma in 39 patients within 6 hours of ROSC and 10 healthy subjects as well as CL content in human heart and brain specimens were quantified using a high-resolution liquid chromatography mass spectrometry method. The quantities of brain-type CL species were correlated with clinical parameters of brain injury severity permitting derivation of a cerebral CL score (C-score) using linear regression. C-score and a single CL species (70:5) were evaluated in patients with varying neurological injury and outcome. Using a rat model of CA, CL was quantified in the plasma and brain of rats using similar methods and results compared with the controls. RESULTS/ANTICIPATED RESULTS: We found that brain and the heart fell on extreme ends of the CL diversity spectrum with 26 species of CL exclusively present in human brain not heart. Nine of these 26 species were present in plasma within 6 hours of ROSC with quantities correlating with greater brain injury. The C-score correlated with early neurologic injury and predicted discharge neurologic/functional outcome. CL (70:5) emerged as a potential point-of-care marker that alone was predictive of injury severity and outcome nearly as well as C-score. Using a rat CA model we showed a significant reduction in hippocampal CL content corresponding to CL released from the brain into systemic circulation. C-score was significantly increased in 10 minute Versus 5 minute no-flow CA and naïve controls. DISCUSSION/SIGNIFICANCE OF IMPACT: CA results in appearance and accumulation of CL in plasma, proportional to injury severity. Quantitation of brain-type CL species in plasma can be used to prognosticate neurological injury within 6 hours after ROSC.

